# Composition of fatty acids in plasma and erythrocytes and eicosanoids level in patients with metabolic syndrome

**DOI:** 10.1186/1476-511X-10-82

**Published:** 2011-05-19

**Authors:** Tatyana P Novgorodtseva, Yulia K Karaman, Natalia V Zhukova, Elena G Lobanova, Marina V Antonyuk, Tatyana A Kantur

**Affiliations:** 1 Vladivostok Branch of the Far Eastern Center of Physiology and Pathology of Respiration of SB RAMN - Institute of Medical Climatology and Rehabilitative Treatment, Russia; 2 Institute of marine biology of name A.V. Zhirmunskogo of the Far East department the Russian academy of sciences, Vladivostok, Russia

## Abstract

**Background:**

Disturbances of the fatty acids composition in plasma and red blood cells and eicosanoid synthesis play an important role in the metabolic syndrome (MS) formation.

**Methods:**

The observation group included 61 people with metabolic syndrome (30 patients with MS and normal levels of insulin, 31 people with MS and insulin resistance - IR). The parameters of carbohydrate and lipid metabolism in blood serum were examined. The composition of nonesterified fatty acids (NEFA), fatty acid (FA) of red blood cells lipids was analyzed by gas-liquid chromatography. Eicosanoids level in MS patients blood serum was studied by enzyme immunoassay.

**Results:**

In MS patients in the absence of glucose-insulin homeostasis disturbances and in patients with IR the accumulation of polyunsaturated fatty acids (18:2 n6, 18:3 n3, 22:4 n6) and lower pool of saturated FA (12:0, 14:0, 16: 0, 17:0) in plasma were discovered. A deficit of polyunsaturated FA (18:3 n3, 20:4 n6) with a predominance of on-saturated FA (14:0, 18:0) in erythrocyte membranes was revealed. In MS patients regardless of the carbohydrate metabolism status high levels of leukotriene B4 and 6-keto-prostaglandin-F1α in serum were found. The development of IR in MS patients leads to increased synthesis of thromboxane A2.

**Conclusion:**

The results revealed a disturbance in nonesterified fatty acids of plasma lipids and red blood cells, eicosanoid synthesis in MS patients. The breach of the plasma and cell membranes fatty acids compositions, synthesis of vasoactive and proinflammatory eicosanoids is an important pathogenetic part of the MS development.

## Background

Metabolic syndrome (MS), which includes a number of systemic clinical and biochemical processes (insulin resistance, abdominal obesity, hypertension, dyslipidemia), attracts the attention of endocrinologists, cardiologists, general practitioners [1-3]. This is due to the large spread of this syndrome in the population (20%), and its value in the development of cardiovascular disease, diabetes mellitus type 2 [1,4]. There are several hypotheses of MS, and the theory of insulin resistance (IR) is main [5,6]. According to some authors the formation of insulin resistance precedes the deficit of essential polyunsaturated fatty acids (PUFA) in the cells. The one of the reasons may be a disturbances of their active receptor (apo B/100) transport of lipoproteins [7-9]. Endogenous fatty acid deficiency in cells leads to changes of phospholipids fatty acid composition and physicochemical properties of plasma membrane, lowering their liquid, breaking functioning of the insulin receptor and glucose transport systems. Logical consequence of receptor transfer of fatty acid (FA) blockade is a compensatory increase in passive absorption of nonesterified free FA (FFA) by cells [10-13]. Cells adaptation to this type of FFA transport activates lipolysis, enhances insulin secretion exponentiating the hyperinsulinemia (HI) formation [14]. In turn, a disturbance of HI autoregulation of insulin receptors further enhances peripheral IR.

Another negative side of physiologically important PUFA in the cells membrane pool depletion is the dysfunction of biologically active metabolites synthesis - oxylipins (eicosanoids: prostaglandins, leukotrienes, thromboxanes), which are the key regulators of endothelial function, immunocompetent cells, platelets [15]. It's proved that the disturbance of the eicosanoids synthesis and it's imbalance in the body cause the chronic inflammation, arterial hypertension, coronary heart disease, atherosclerosis, diabetes mellitus [16,17]. Chain of successive violations, starting from the pathology of FA transport receptor, leading to cellular deficiency of essential fatty acids and eicosanoid synthesis disturbance, creating a vicious circle forming MS. However, nowaday there is no clear evidence supporting the pathogenetic role of fatty acids and disturbances of their transport and eicosanoids synthesis dysfunction in the metabolic syndrome mechanisms.

Purpose: to study the fatty acid composition in plasma and red blood cells and the level of oxylipins in MS patients blood with different glucose-insulin homeostasis, to establish the role of fatty acids and their metabolites in the MS pathogenesis.

## Materials and methods

76 people (30 men, 46 women) aged 21 to 69 years participated in a study with informed consent. Criteria proposed by the American Heart Association were used for the MS diagnosis [3]. Depending on the availability of MS components and changes in glucose-insulin homeostasis 76 people were divided into the following groups: Group 1 (control) consisted of 15 persons without MS components, Group 2 included 30 MS patients with normal levels of insulin, the third group comprised 31 persons with diagnosed MS and IR.

The study of carbohydrate metabolism includes the determination of the glucose content in the blood serum fasting and 2 hours after oral glucose load, insulin levels by immunosorbent method (kits of «DRG - diagnostics» firm, Germany), calculated HOMA index (fasting insulin level, mU/ml × level fasting plasma glucose, mmol/l/22.5). The serum lipid spectrum was studied by the content of cholesterol, triglycerides (TG), high density lipoprotein cholesterol (HDL) in serum with biochemical analyzer Photometer FM 750 (Germany), using sets of firm "Olvex" (Russia). Results were expressed in mmol/liter. The concentration of low density lipoproteins cholesterol (LDL) and very low (VLDL) density were calculated by the formula: LDL = total cholesterol - (HDL + VLDL), VLDL = TG/2,2, the results were expressed in mmol/liter [18].

The extraction of lipids from erythrocytes was performed by a modified method of Bligh and Dyer [19]. Gas-liquid chromatography of FA methyl ethers was performed on gas-liquid chromatograph "Shimadzu-9A" (Japan) with a flame ionization detector. Methyl ethers of erythrocytes FA lipids were obtained by the method of Carreau and Dyubak [20] and purified by thin-layer chromatography. FA methyl ethers were analyzed by capillary columns. The Chromaton N-AW-DMCS 0,100-0,125 was used as carrier, 5% Silar-5 CP, FFAP - as the liquid phase. The evaporation temperature was 245°C. The separation temperature was 210°C. Helium was the gas-carrier with linear velocity - 20 cm/sec. The identification was performed using standard mixtures of FA and the values of the equivalent chain length [21]. Quantitative calculations were performed using the standard software of data processing systems Chromatopak-CR 3A. Results were expressed in relative % of total FA. The level of eicosanoids (thromboxane B2, 6-keto-prostaglandin F1α, leukotriene B4) in the blood was studied by enzyme immunoassay (kits of Amersham Biosciences firm, UK).

Statistical data processing was performed using the methods of descriptive statistics: the arithmetic mean, the standard error of arithmetic mean (M ± m), the criteria for significant differences (t) Student.

## Results

Expressed clinical and metabolic changes specific for this syndrome was revealed in MS patients: increased body mass index, ratio of waist and hips (W/H), increased blood pressure, increased in the LDL-C. In both groups MS patients had increased level of proinflammatory cytokine (TNF-α) in blood (Table 1).

**Table 1:** Clinical and biochemical characteristics of patients with metabolic syndrome

Parameters	Control group, n = 15	2 group (with MS), n = 31
BMI	21,97 ± 0,65	***31,55 ± 1,79
Waist circumference,cm	70,45 ± 1,55	***95,76 ± 3,98
Hip circumference,cm	98,63 ± 1,88	***116,07 ± 3,40
Waist-hip ration, a.u.	0,71 ± 0,01	***0,82 ± 0,01
Systolic arterial pressure, mmHg	105 ± 2	***142 ± 2
Diastolic arterial pressure, mmHg	65 ± 2	***88 ± 2
Fasting plasma glucose, mmol/l	4,06 ± 0,31	**5,80 ± 0,22
Glucose after oral load, mmol/l	4,32 ± 0,39	*6,52 ± 0,65
Insulin, mU/ml	8,08 ± 1,04	***18,50 ± 2,19
HOMA index	1,50 ± 0,21	***4,50 ± 0,66
TC, mmol/l	4,44 ± 0,21	**5,80 ± 0,29
ТG, mmol/l	0,68 ± 0,07	*1,79 ± 0,14
HDL, mmol/l	1,41 ± 0,11	1,14 ± 0,06
LDL, mmol/l	2,72 ± 0,22	*3,88 ± 0,28

Abbreviation: BMI, body mass index (calculated as weight in kilograms divided by height in meters squared);

Note: tabl. 1, 2, 3 (*) - Statistically significant differences linked to the control group: * - p < 0.05, ** - p < 0.01, *** - p < 0,001.

Qualitative composition of the NEFA in examined groups is presented by 31 components of individual fatty acids with carbon chain lengths from C12 to C24, as with the even and odd number of carbon atoms, normal and isostructure, saturated, polyunsaturated and monoenic. Composition of the main FA in plasma and FA in red blood cells lipids in MS patients is presented in Table 2. Analysis of the quantitative composition NEFA showed that MS patients in group 2 in compare with the control group had a decrease of individual saturated FA levels: lauric (12:0), myristic (14:0, p < 0.05), palmitic (16:0 p < 0,01), margarine (17:0, p < 0.05) (Table 2). Among the isostructure acid of the relative content of 16:0-i (p < 0,01) was almost 3 times decreased. Since the relative amounts of saturated fatty acids is reduced the content of polyunsaturated FA is increased. Proportion of linoleic (18:2 n6) and α-linolenic (18:3 n3) acids increased twofold (p < 0,01), which was reflected in increased total index n6 FA - Σ n6 FA. Integrated parameter of changes in the FA variety was the index of unsaturation (US), calculated as the sum of products of double bonds in each of the FA with its relative percentage. This parameter was increased (p < 0,05). In the third group (MS and IR patients) vector of changes in the NEFA composition was comparable with the same in group 2. The obtained results show that patients with the absence of glucose-insulin homeostasis disturbances and patients with severe IR had quite pronounced changes in the NEFA composition, caused by a violation of the transfer and absorption of the saturated and polyunsaturated FA in the blood cells [22]. The consequence of these processes can be deficit of essential polyunsaturated acids in the cells.

**Table 2:** The content of NEFA in plasma and FA in erythrocyte lipids in patients with metabolic syndrome

Fatty acids, %	Control group, n = 15	2 group (with MS), n = 31
12:0	0,65 ± 0,10 0,40 ± 0,01	*0,45 ± 0,07 *0,2 ± 0,07
14:0	2,96 ± 0,21 0,65 ± 0,04	2,91 ± 0,17 *0,76 ± 0,02
15:0	0,88 ± 0,11 0,28 ± 0,02	0,78 ± 0,13 0,29 ± 0,01
16:0	33,76 ± 1,25 23,37 ± 0,32	*30,79 ± 1,25 24,03 ± 0,37
16:0-i	1,62 ± 0,35 0,40 ± 0,01	**0,41 ± 0,07 0,50 ± 0,01
16:1n7	2,01 ± 0,27 0,8 ± 0,03	2,17 ± 0,32 **0,64 ± 0,03
17:0	1,25 ± 0,19 0,45 ± 0,04	*0,92 ± 0,03 0,43 ± 0,03
17:1	0,3 ± 0,01 0,37 ± 0,025	0,35 ± 0,08 ***0,16 ± 0,02
18:0	31,32 ± 5,07 17,23 ± 0,29	29,15 ± 4,85 ***22,8 ± 0,9
18:0-i	0,25 ± 0,08 0,80 ± 0,01	Traces **0,57 ± 0,07
18:2n6	7,53 ± 1,68 13,74 ± 0,53	*11,13 ± 2,21 *11,98 ± 0,34
18:3n6	0,13 ± 0,03 0,20 ± 0,01	Traces 0,21 ± 0,02
18:3n3	0,51 ± 0,11 0,20 ± 0,01	*0,82 ± 0,17 Traces
20:0	0,89 ± 0,09 0,18 ± 0,02	0,74 ± 0,11 **0,3 ± 0,03
20:3n9	0,60 ± 0,21 0,56 ± 0,24	**0,36 ± 0,06 *0,88 ± 0,18
20:4n6	0,36 ± 0,06 12,13 ± 0,24	0,41 ± 0,06 ***10,14 ± 0,35
20:5n3	0,66 ± 0,11 1,08 ± 0,11	0,45 ± 0,07 **1,58 ± 0,11
22:1	Traces 0,25 ± 0,02	Traces ***0,67 ± 0,10
22:4n6	1,74 ± 0,12 2,15 ± 0,11	1,94 ± 0,11 **1,58 ± 0,09
Σ n6	7,77 ± 1,71 30,1 ± 0,6	*11,42 ± 2,27 ***25,7 ± 0,67
Σ n3	1,01 ± 0,18 9,98 ± 0,78	1,21 ± 0,22 9,94 ± 0,47
Unsaturation index	38,41 ± 7,30 155,98 ± 1,33	*49,08 ± 8,83 **133,16 ± 6,17

Note: in numerator - the nonesterified FA in plasma, in denominator - FA in erythrocyte lipids

To confirm this assumption red blood cells FA lipids composition in MS patients has been studied. In the erythrocytes FA lipids composition 37 individual fatty acids, saturated and polyunsaturated monoenic, normal, and isostructure with chain length from C12 to C22, as with the even and odd number of carbon atoms (Table 2) were identified. In the quantitative composition of erythrocyte FA lipids in surveyed MS patients significant changes compared with the control group were revealed. In MS patients without IR in erythrocytes the proportion 20:0 FA was increased, monoenic (16:1 n7, 17:1) and polyunsaturated (18:3 n3, 20:4 n6) fatty acids were reduced. An increase in the relatively level of Mead acid (20:3 n9) was revealed. Compensatory synthesis of Mead acid occurs with a deficit of polyunsaturated acids family n6 and n3 [7]. In the group 3 the accumulation of myristic, stearic and arahidonic (20:0) acids was revealed on the background of significant reduction in the proportion of essential linoleic acid (18:2 n6), 18:4 n3, arachidonic (20:4 n6) and 22:4 n6 polyunsaturated FA, increasing 20:3 n9. In both groups the total parameter set-Σ n6 (p < 0.05) and US (p < 0,01) was reduced.

The results testify the modifications of the free fatty acids composition in blood plasma and erythrocyte fatty acids in patients with MS components. The causes of PUFA accumulation in plasma simultaneously with their deficiency in cells may be a disturbances in the cells receptor apparatus responsible for the active capture of the FA as part of lipoproteins. Changing of the composition of fatty acid (FA) in the cells membranes, mainly in the downward the number of esterified essential polyunsaturated fatty acids into the phospholipids leads to decreasing the negative charge of the membrane, increasing its microviscosity, activation of proinflammatory eicosanoids synthesis and increased sensitivity of arteries smooth muscle cells to the vasoconstrictors effects [23,24]. Presented disturbances are the main pathogenetic factors of cardiovascular disease formation (hypertension, stroke, myocardial infarction). The observed decrease of arachidonic acid in red blood cells indicates on a disturbances in eicosanoids cycle and increased oxylipins synthesis with expressed vasoconstrictor (thromboxane A2) and proinflammatory (leukotriene B4) properties.

The research of eicosanoids levels in blood serum of MS patients showed that patients from 2 and 3 groups had increased concentrations of 6-keto-prostaglandin F1α (p < 0,001) and leukotriene B4 (p < 0.001) in contrast with the control group (Table 3). In the third group increased level of thromboxane B2 (p < 0,001) was shown, which was not noted in the 2 group. Elevated levels of leukotrienes, which are the strongest mediators of allergic and inflammatory processes as well as a high content of TNF-α in the MS patients blood indicate on the activation of inflammatory reactions [25]. The observed excess of 6-keto-prostaglandin F1α, which is a potent vasodilator [26] in MS patients suggests the launch of the compensatory mechanisms that support the balance preservation between the formation of pro-and anti-oxylipins. However, the attempt of the organism to maintain a dynamic equilibrium between leukotrienes and prostaglandins has its limitations. Identified disturbances can be a decisive step in the launch of the mechanisms of cardiovascular diseases, diabetes and other pathologies developing in MS patients. This was confirmed by the overproduction of thromboxane B2 in patients with MS complicated by insulin resistance, which indicates a connection of MS pathogenetic mechanisms such as vasoconstriction, hypercoagulation, which are amplifying disturbances in the vascular wall dysfunction, leading to the increased cells resistance to insulin [16].

**Table 3:** The level of eicosanoids in blood of metabolic syndrome patients

Eicosanoids	Control group, n = 15	2 group (with MS), n = 31
6-keto-prostaglandin F1α, pg/ml	20,75 ± 1,004	***38,24 ± 2,94
leukotriene B4, pg/ml	149,67 ± 3,28	***217,37 ± 8,58
thromboxane B2, pg/ml	245,73 ± 21,12	*301,33 ± 9,62

Significant role in the modification of the FA and the formation of a substrate for the synthesis of eicosanoids also belongs to the delta-5 and 6-desaturases. However, in our work activity desaturases has not been studied. Need for further research into the causes modifications of the FA in the pathogenesis of MS.

Thus, the development of MS is accompanied by a modification of nonesterified and esterified FA in plasma and blood cells. One of the reasons for FA composition changes may be a disturbances of their active transport. This leads to the changes in the cell membranes structure, a decrease of insulin-dependent glucose transporter functional activity, disruption of oxylipins synthesis and imbalance between pro-and anti-inflammatory, vasoconstrictor and vasodilatory eicosanoids. Displacement of the dynamic equilibrium of the cytoprotective and cytotoxic eicosanoids biosynthesis in favor of the latter, the disruption of insulin receptors initiate the pathogenetic mechanisms of development and progression of metabolic complications is a major component of the cardiovascular diseases, diabetes formation. The breach of the fatty acids of plasma and cell membranes, synthesis of vasoactive and proinflammatory eicosanoids is the cause of MS. These findings reveal an important role of fatty acids and their metabolites in the pathogenesis of the metabolic syndrome, which should be considered in the design and choice of preventive and therapeutic interventions directed to preventing or eliminating the identified irregularities in lipid-transfer, cyclooxygenase and lipoxygenase systems.

## Competing interests

The authors declare that they have no competing interests.

## Authors' contributions

YK, EL, TK and NZ participated in the design of the study, performed the statistical analysis and drafted the manuscript, contributed to acquisition of data and its interpretation. MA contributed to conception and design of the statistical analysis. TN conceived of the study, participated in its design, coordination and helped to draft the manuscript. All authors read and approved the manuscript.
